# Tobacco products and sensory health: An assessment of taste and smell disorders using 2021 NHIS data

**DOI:** 10.18332/tid/181289

**Published:** 2024-02-08

**Authors:** Rahaf H. Bin Hamdan, Waad R. AlAmri, Muath A. Aldosari

**Affiliations:** 1 Dental Administration, Ministry of Health, Al Qurayyat, Saudi Arabia; 2 Periodontics and Community Dentistry, College of Dentistry, King Saud University, Riyadh, Saudi Arabia; 3 Ministry of Health, Bisha, Saudi Arabia; 4 Oral Health Policy and Epidemiology, Harvard School of Dental Medicine, Harvard University, Boston, United States; 5 Health Policy and Health Services Research, Goldman School of Dental Medicine, Boston University, Boston, United States

**Keywords:** tobacco, e-cigarettes, smell disorder, taste disorder, NHIS

## Abstract

**INTRODUCTION:**

This study aims to assess the relationship between use of tobacco products and taste and smell disorders.

**METHODS:**

A secondary dataset analysis of cross-sectional data from the National Health Interview Survey (NHIS) 2021 survey cycle, a nationally representative annual cross-sectional interview of non-institutionalized US civilians, was used. Participants' senses of smell and taste are assessed using self-reported questions. Participants were categorized into five groups based on their tobacco use: non-tobacco users; cigarettes only; e-cigarettes only; cigar, pipe, or smokeless tobacco; and poly-tobacco product users. Disorders were defined as any self-reported difficulty in smelling, tasting, or reporting unpleasant odors or persistent tastes. Weighting procedures were used to estimate the national prevalence of taste, smell, and other disorders, stratified by tobacco products used. Adjusted logistic regression models were used to determine the association between tobacco products used and taste or smell compared to non-tobacco users.

**RESULTS:**

A total of 40.2 million US adults reported experiencing smell or taste disorders. Nearly one in 10 adults reported a taste disorder (9.8%), and 13.4% indicated a smell disorder. The prevalence of taste or smell disorder was higher among females (17.5%), Hispanics (19.5%), individuals identified as ‘other’ racial minorities (21.1%), and lower income groups (21%). Compared to non-tobacco users, the highest odds of experiencing smell or taste disorders were among poly-tobacco product users (adjusted odds ratio, AOR=1.44; 95% CI: 1.31–1.58), followed by e-cigarette-only users (AOR=1.38; 95% CI: 1.02–1.87), cigarette-only smokers (AOR=1.17; 95% CI: 1.04–1.32), and users of cigars, pipes, or smokeless tobacco (AOR=1.15; 1.00; and 1.33; respectively).

**CONCLUSIONS:**

Tobacco product use was associated with an increased risk of smell and taste disorders. The rising use of e-cigarettes among adolescents and young adults is particularly concerning given the limited understanding of the sensory effects of e-cigarettes and their growing popularity among younger populations. The study findings highlight the need for interventions aimed at reducing tobacco use of all kinds.

## INTRODUCTION

Tobacco use is a leading cause of preventable morbidities and mortalities in the United States (US)^1^. Since the Surgeon General’s report on smoking in 1964, tobacco use has been linked to numerous health problems, including cardiovascular disease, respiratory disease, and various cancers^1-3^. According to the Centers for Disease Control and Prevention (CDC), cigarette smoking alone causes approximately 480000 deaths each year in the US, accounting for about one in five deaths annually^2^.

Although the prevalence of cigarette smoking has declined, the prevalence of e-cigarette consumption has risen^4,5^. One of the appeals of e-cigarettes is that they often combine nicotine with aromas and flavors. While some use these products as smoking cessation tools, they do not only carry potential risks but also pose a significant threat of leading users back to traditional tobacco cigarette smoking^6^. Results from a national survey have shown an increasing trend of non-cigarette tobacco product use among adolescents, including e-cigarettes and hookah^7^.

All tobacco products, even non-cigarette ones, contain a variety of harmful chemicals that can lead to significant health issues^8^, including the sensory health of individuals. Physiologically, repeated smoking exposure results in a loss of odor sensitivity and olfactory recognition, which subsequently diminishes the capacity for sensory cell production^9^. Taste disturbances arise from changes in the vascularization, shape, and number of taste buds due to tobacco smoking, leading to gustatory disturbances^9^. Moreover, growing concerns about the potential impact of non-cigarette tobacco products on our senses of taste and smell should not be underestimated^10^. These vital senses not only enhance our culinary experiences but also provide us with the ability to detect potentially harmful substances^10,11^. In addition to affecting smell and taste, e-cigarette use is associated with a higher plaque index, probing depth, clinical attachment loss, and peri-implant bone loss, compared to traditional smokers, as evidenced by a systematic review of the detrimental effects of e-cigarettes on oral health^12^.

To date, much of the research on the relationship between tobacco use and taste and smell disorders has centered on cigarette smoking. However, there is limited research on the association between taste and smell disorders and the use of non-cigarette tobacco products. In this study, we aim to explore the association between the use of various tobacco products and taste and smell disorders, drawing on data from the National Health Interview Survey (NHIS).

## METHODS

### Study design and population

The study is a secondary dataset analysis of NHIS data. The NHIS is an annual cross-sectional household interview survey targeting the civilian, non-institutionalized population of the US. The data are provided separately for sample children (aged 0–17 years) and sample adults (aged ≥18 years). The NHIS is a publicly available, de-identified dataset and stands as one of the essential resources for health-related data. It is administered by the National Center for Health Statistics (NCHS), a division of the CDC, to monitor and track the nation’s progress toward achieving health objectives^13^. We utilized the data of the sample adults from the 2021 cycle, with a total sample size of 29482 participants (a 50.9% participation rate). The NHIS had received ethical approval from both the Research Ethics Review Board of the NCHS and the U.S. Office of Management and Budget. All participants provided verbal consent before participating in the interview.

### Definition of smell and taste disorder

Participants’ senses of smell and taste were assessed using a series of self-reported questions. To evaluate smell function, adults were asked: ‘During the past 12 months, have you had difficulty with your sense of smell or ability to detect odors?’. They could choose from five responses, ranging from ‘no difficulty’ to ‘cannot smell at all’. Another question inquired about the experience of phantom unpleasant odors, asking: ‘During the past 12 months, did you sometimes smell an unpleasant, bad, metallic, or burning odor when nothing was there?’. A participant was considered to have a smell disorder if they reported any difficulty in smelling or any experience of phantom unpleasant odors.

To assess taste function, participants were asked: ‘During the past 12 months, have you had difficulty with your ability to taste sweet, sour, salty, or bitter foods and drinks?’. The five possible responses ranged from ‘no difficulty’ to ‘cannot taste at all’. The study also inquired about the persistence of an unwanted taste or sensation in the mouth with the question: ‘During the past 12 months, have you had an unwanted taste or other sensation in your mouth that doesn’t go away?’. A taste disorder was defined as any reported difficulty in tasting or the presence of an unwanted, persistent taste. Finally, participants who reported impairment in either sense were categorized as having a smell or taste disorder.

Definition of tobacco use and confounding factors

To determine the smoking status of participants in the NHIS, we categorized individuals into five distinct groups. Non-tobacco users were defined as those who reported no current or past use of any tobacco products. Those who exclusively used cigarettes, without any current or past use of other tobacco products, were placed in the ‘cigarettes only’ category. The ‘e-cigarettes only’ group included individuals who exclusively used e-cigarettes without any current or past consumption of other tobacco products. Participants who reported using only cigars, pipes, or smokeless tobacco, without any current or past use of cigarettes or e-cigarettes, were categorized as ‘cigar, pipe, or smokeless tobacco’. ‘Poly-tobacco product users’ were defined as those who reported current or past use of multiple tobacco products. Other independent variables included in the analysis were age (18–24, 25–44, 45–64, ≥65 years), sex (male or female), race (non-Hispanic White, non-Hispanic Black, Hispanic, non-Hispanic Asian, other), and income in Federal Poverty Level (% of FPL: <100, 100–199, 200–299, ≥300).

### Statistical analysis

We first determined the frequency distribution of sociodemographic characteristics for our sample. Then, using weighting procedures to account for the sample design, we estimated the weighted prevalence and national counts of the presence of taste, smell, or any disorders broken down by sociodemographic characteristics. We applied the chi-squared test to assess the association between sociodemographic factors and the prevalence of these disorders. Following that, we reported the overall prevalence of each disorder by smoking status, along with the corresponding 95% confidence interval (95% CI).

Lastly, we used logistic regression models to present both crude (OR) and adjusted odds ratio (AOR), accompanied by the respective 95% CI, for the presence of taste or smell disorders among various tobacco user groups compared to non-smokers. Age, sex, race, and income were considered potential confounders in the relationship between smoking status and the occurrence of disorders based on a theoretical framework illustrated in the constructed Directed Acyclic Graph (DAG) (Supplementary file Figure 1). We conducted a sensitivity analysis to determine any potential influence on the results if former tobacco users were categorized as never tobacco users, investigated the association between tobacco use and co-occurring smell and taste disorders, and the interaction between tobacco products and sex on sensory disorders. The alpha (set at 0.05) was two-tailed to test the relationship in both directions, and all statistical analyses were carried out using Stata/BE 17.0.

## RESULTS

A total of 40.2 million US adults, representing 16.5% of the population, reported experiencing smell or taste disorders (Table 1). A significant portion of this group indicated a smell disorder (13.4%), while nearly one in 10 adults reported a taste disorder (9.8%). The prevalence of taste disorders was notably higher in females (10.7%) than in males (8.8%) (p<0.01). A similar trend was observed for smell disorders, with 14.3% of females and 12.5% of males reporting having issues (p<0.01). Non-Hispanic Asians had the lowest prevalence of smell or taste disorders (10.3%), whereas the highest prevalence was observed among Hispanics (19.5%) and other racial minorities, including native and multi-racial groups (21.1%) (p<0.01). Lower income groups exhibited the highest prevalence of both taste and smell disorders (p<0.01), with a clear decline in prevalence as income levels increased.

**Table 1:** Demographic characteristics and prevalence of smell, taste, and smell or taste disorders, among US adults, aged ≥18 years, who reported their smoking status and responded to taste and smell questions in the 2021 National Health Interview Survey (NHIS)

*Characteristics*	Overall n (%*)	*Taste disorder*		*Smell disorder*		*Smell or Taste disorder*		*p*
		%*	*Weighted US population N (in thousands)*	%*	*Weighted US population N (in thousands)*	%*	*Weighted US population N (in thousands)*	
**Overall**	28523 (100)	9.8	23906	13.4	32816	16.5	40234	
**Sex**								
Male	12950 (48.4)	8.8	10411	12.5	14771	15.5	18224	<0.01
Female	15573 (51.6)	10.7	13491	14.3	18045	17.5	22006	
**Age** (years)								
18–24	1767 (11.5)	8.6	2417	13.0	3638	15.5	4339	<0.01
25–44	8819 (34.2)	10.4	8685	13.7	11454	16.5	13754	
45–64	9288 (32.1)	10.4	8172	14.0	10996	17.2	13452	
≥65	8651 (22.2)	8.5	4631	12.4	6728	16.1	8689	
**Race**								
Non-Hispanic White	19141 (63.2)	9.6	14787	13.5	20771	16.5	25354	<0.01
Non-Hispanic Black	2993 (11.5)	9.1	2549	11.4	3204	14.4	4038	
Hispanic	3910 (16.8)	11.9	4863	16.2	6626	19.5	7992	
Non-Hispanic Asian	1733 (5.9)	6.3	905	7.4	1058	10.3	1487	
Other	748 (2.7)	12.4	802	17.9	1156	21.1	1363	
**Income** (% of FPL)								
<100	2769 (9.8)	12.6	3014	17.5	4184	21.0	5025	<0.01
100–199	4937 (17.4)	11.6	4942	14.7	6242	18.7	7941	
200–299	4630 (16.4)	10.5	4205	14.5	5810	18.0	7205	
≥300	16189 (56.4)	8.5	11745	12.0	16580	14.6	20063	

* The sample counts are unweighted while percentages are weighted to account for complex survey design. FPL: Federal Poverty Level.

**Figure 1:**
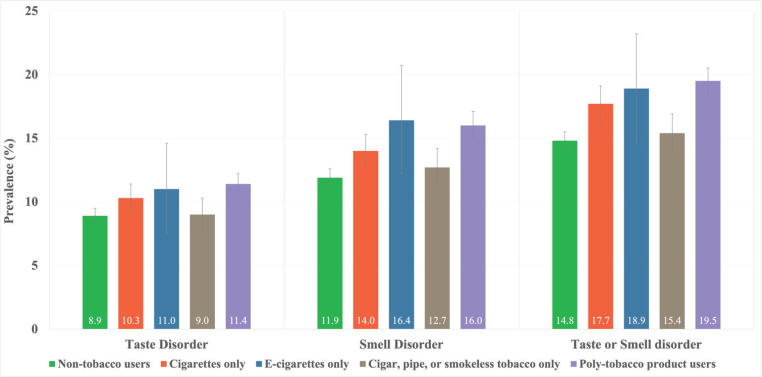
Prevalence of smell, taste, and smell or taste disorders, by tobacco use groups among US adults, aged ≥18 years, who reported their smoking status and responded to taste and smell questions in the 2021 National Health Interview Survey (NHIS)

The observed prevalence of smell or taste disorders among non-tobacco users was 14.8% (95% CI: 14.0–15.5), which is below the national average of 16.5% (95% CI: 15.9–17.1) (Figure 1, Table 2). Among tobacco users, those who consumed e-cigarettes exhibited the highest prevalence of a smell disorder at 16.4% (95% CI: 12.2–20.7) and the second-highest prevalence of a taste disorder at 11.0% (95% CI: 7.5–14.6), surpassed only by poly-tobacco product users with16.0% (95% CI: 15.1–17.1) for smell disorder, and 11.4% (95% CI: 10.5–12.2) for taste disorder. Poly-tobacco product users reported the highest prevalence of either smell or taste disorder at 19.5% (95% CI: 18.4–20.5), followed by e-cigarette users at 18.9% (95% CI: 14.6–23.2), cigarette-only users at 17.7% (95% CI: 16.2–19.1), and users of other tobacco products (such as cigars, pipes, or smokeless tobacco) at 15.4% (95% CI: 13.8–16.9).

**Table 2:** Prevalence of smell, taste, and smell or taste disorders by tobacco use groups among US adults, aged ≥18 years, who reported their smoking status and responded to taste and smell questions in the 2021 National Health Interview Survey (NHIS)

*Smoking status*	*Taste disorder (N=29444)*		*Smell disorder (N=29421)*		*Smell or taste disorder (N=29403)*	
	%*	*95% CI*	%*	*95% CI*	%*	*95% CI*
Total US adults	9.8	(9.3–10.2)	13.5	(12.9–14.0)	16.5	(15.9–17.1)
Non-tobacco users	8.9	(8.3–9.5)	11.9	(11.2–12.6)	14.8	(14.0–15.5)
Cigarettes only	10.3	(9.2–11.4)	14.0	(12.8–15.3)	17.7	(16.2–19.1)
E-cigarettes only	11.0	(7.5–14.6)	16.4	(12.2–20.7)	18.9	(14.6–23.2)
Cigar, pipe, or smokeless tobacco only	9.0	(7.7–10.3)	12.7	(11.2–14.2)	15.4	(13.8–16.9)
Poly-tobacco product users	11.4	(10.5–12.2)	16.0	(15.1–17.1)	19.5	(18.4–20.5)

* Weighted percentages.

**Figure 2:**
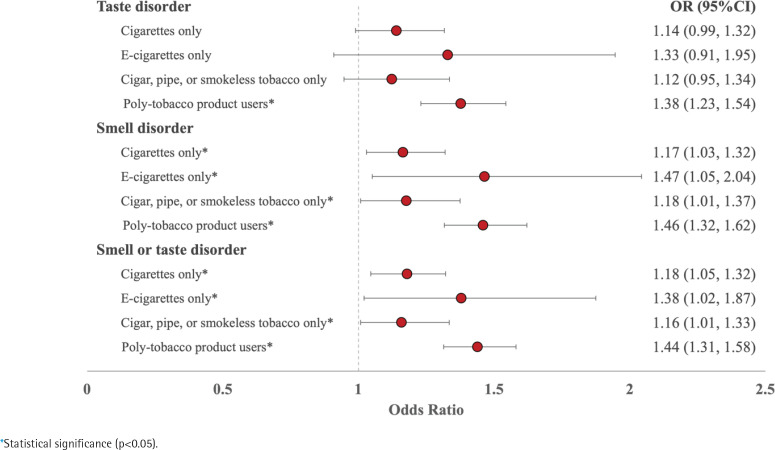
Adjusted odds ratios (AORs) and 95% confidence intervals (95% CIs) of experiencing taste and smell disorders compared to non-tobacco users among US adults, aged ≥18 years, who reported their smoking status and responded to taste and smell questions in the 2021 National Health Interview Survey (NHIS), adjusting for gender, age, race, and income level

After adjusting for sociodemographic factors, taste disorders were not statistically significantly higher in single tobacco users compared to non-users among US adults, except for poly-tobacco product users who had 1.37 times the odds of reporting a taste disorder (95% CI: 1.23–1.54) (Figure 2, Table 3). However, all tobacco groups reported smell disorders, with e-cigarette-only users having the highest odds compared to non-tobacco users (AOR=1.47; 95% CI: 1.05–2.04). Likewise, all tobacco groups had higher odds of experiencing either smell or taste disorders than non-users. Poly-tobacco product users had 1.44 times the odds compared to non-users (95% CI: 1.31–1.58), followed by e-cigarette-only users with 1.38 times the odds (95% CI: 1.02–1.87), users of cigars, pipes, or smokeless tobacco with 1.15 times the odds (95% CI: 1.00–1.33), and cigarette-only users (OR=1.17; 95% CI: 1.04–1.32).

**Table 3:** Logistic regression models of associations between smoking status and smell or taste disorders, taste disorder, and smell disorder among US adults, aged ≥18 years, who reported their smoking status and responded to taste and smell questions in the 2021 National Health Interview Survey (NHIS)

*Tobacco product*	*Taste disorder*		*Smell disorder*		*Smell or Taste disorder*	
	*OR (95% CI) (N=29444)*	*AOR (95%CI) (N=29442)*	*OR (95%CI) (N=29421)*	*AOR (95%CI) (N=29419)*	*OR (95%CI) (N=29403)*	*AOR (95%CI) (N=29401)*
Non-tobacco user ®	1	1	1	1	1	1
Cigarettes only	1.17* (1.02–1.34)	1.14 (0.98–1.31)	1.20* (1.06–1.36)	1.16* (1.02–1.31)	1.23* (1.10–1.38)	1.17* (1.04–1.32)
E-cigarettes only	1.26 (0.87–1.81)	1.33 (0.90–1.94)	1.45* (1.06–1.98)	1.47* (1.05–2.04)	1.34* (1.00–1.78)	1.38* (1.02–1.87)
Cigar, pipe, or smokeless tobacco only	1.00 (0.84–1.19)	1.12 (0.94–1.33)	1.07 (0.92–1.24)	1.17* (1.00–1.37)	1.04 (0.91–1.19)	1.15* (1.00–1.33)
Poly-tobacco product users	1.31* (1.18–1.45)	1.37* (1.23–1.54)	1.41* (1.28–1.55)	1.46* (1.31–1.62)	1.39* (1.27–1.51)	1.44* (1.31–1.58)

AOR: adjusted odds ratio; adjusted for age, gender, race, and income. ® Reference category.

* Statistical significance (p<0.05).

In the sensitivity analyses, where former tobacco users were classified as never tobacco users, we reported the weighted prevalence of disorders by tobacco use. The results showed that e-cigarette users 21.1% (95% CI: 17.3–24.9) and poly-tobacco product users 22.5% (95% CI: 19.2–25.7) had the highest prevalence of taste or smell disorders compared to other groups (Supplementary file Table 1). Additionally, the adjusted odds ratio of experiencing taste or smell disorders was significant only among e-cigarette users (AOR=1.42; 95% CI: 1.13–1.80) and poly-tobacco product users (AOR=1.47; 95% CI: 1.21–1.78) (Supplementary file Table 2). The adjusted OR of co-occurring taste and smell disorders was significant only among poly-tobacco product users (AOR=1.48; 95% CI: 1.29–1.71) (Supplementary file Table 3). Further analysis including an interaction term between tobacco products and sex revealed that, compared to non-tobacco users, both male and female poly-tobacco product users had significantly increased odds of experiencing taste disorders (male AOR=1.33; 95% CI: 1.11–1.60; female AOR=1.49; 95% CI: 1.27–1.74), smell disorders (male AOR=1.53; 95% CI: 1.29–1.81; female AOR=1.43; 95% CI: 1.25– 1.65), and combined smell or taste disorders (male AOR=1.45; 95% CI: 1.25–1.67; female AOR=1.48, 95 % CI: 1.30–1.68) (Supplementary file Table 4).

## DISCUSSION

It is well-known that smoking can cause various health problems, including respiratory and cardiovascular diseases and cancer^8^. However, the impact of tobacco on other aspects of quality of life, such as smell and taste, has been less studied. In this study, we utilized data from the 2021 NHIS national dataset to examine the relationship between the consumption of various tobacco products and disorders of taste and smell. The results indicate that consuming any tobacco product is associated with increased odds of smell and taste disorders, with e-cigarette-only and poly-tobacco product users facing the highest odds of experiencing these sensory disorders. Notably, non-tobacco users also reported some level of smell or taste disorders, suggesting that factors other than smoking may contribute to these conditions.

A key finding of our study is that e-cigarette users face the highest odds of developing sensory disorders compared to other groups. The review of Johnson et al.^14^ demonstrated that little is known about the sensory effects of e-cigarettes. However, as more nicotine is added to e-cigarette liquids, users increasingly perceive irritation and bitterness over time^14^. This revelation heightens concerns about the potential health risks tied to e-cigarette use, especially given the growing trend of use among adolescents and young adults^11^. Nonetheless, it is crucial to interpret these results with caution due to the smaller sample size and broad confidence intervals among e-cigarette users. This smaller sample may have impacted the precision of our findings.

As demonstrated in previous research, smell disorders are more prevalent among smokers than taste disorders^15,16^. Every smoking category showed an increased likelihood of experiencing these sensory disorders. This suggests that the risk is not limited to combustible tobacco products, such as cigarettes, but also encompasses e-cigarettes and other tobacco products, including cigars, pipes, and smokeless tobacco. This is consistent with the study of Pullicin et al.^17^ which observed an increase in perceived irritation and bitterness and a decrease in perceived sweetness when nicotine was added to e-cigarette aerosols. On the other hand, a systematic review by Ralho et al.^12^ found that while e-cigarette smokers have a higher susceptibility to changes in oral biological tissues compared to former smokers or never smokers, e-cigarettes are less harmful than conventional cigarettes. In our sensitivity analysis, we assessed how classifying former users as never tobacco users could impact the results. We found that smoking cigarettes, cigars, pipes, or using smokeless tobacco products, did not show higher odds of taste or smell disorders when former users were categorized as never tobacco users. This suggests that the health impacts of past tobacco use remain significant even for those who have quit. Such findings underscore the need for further research to determine how previous tobacco use might influence sensory function after cessation.

COVID-19 has highlighted the significance of smell and taste sensations in one’s quality of life. One study pinpointed a substantial quality-of-life and personal safety burden due to olfactory deficits in subjects with COVID-19^18^. Smell and taste disorders can profoundly impact a person’s quality of life, leading to a loss of appetite, weight loss, and depression^19,20^. Thus, understanding the risk factors for smell and taste disorders, including smoking, is essential for identifying and managing these conditions.

### Strengths and limitations

One of the strengths of our study is that the NHIS is a large, nationally representative survey. This provides a substantial sample size that allows for the analysis of associations with reasonable statistical power, enhancing the generalizability of our findings to the broader US population. Moreover, the NHIS gathers detailed information on a wide array of health-related variables, including tobacco use, taste and smell disorders, and demographic characteristics, enabling adjustment for potential confounders in our analysis. However, the NHIS is based on self-reported data, which might introduce recall bias or social desirability bias, potentially resulting in the under-reporting or over-reporting of tobacco use and other variables. As a cross-sectional survey, the NHIS does not facilitate the assessment of temporal relationships between tobacco use and disorders of taste and smell. While pre-specifying the chosen confounders in this analysis strengthens its rigor by minimizing bias, some residual confounders were unmeasured or unaccounted for, influencing the observed relationship that cannot be entirely ruled out. Additionally, the survey lacks data on exposure to specific toxicants in non-cigarette tobacco products, constraining our ability to evaluate the potential mechanisms driving the observed associations. The generalizability of our findings to other countries and populations should be cautiously considered. Cultural and environmental differences can alter the interplay between confounders and taste or smell disorders, potentially limiting the applicability of results obtained in this specific context. While the NHIS serves as a valuable data source for studying the relationship between taste and smell disorders and the use of non-cigarette tobacco products, it is crucial to bear its limitations in mind when interpreting our findings. Future research incorporating more detailed measures of taste and smell disorders and exposure to tobacco toxicants may offer clearer insights into the relationships between these variables.

## CONCLUSIONS

The study suggests that smoking, encompassing the use of e-cigarettes and other tobacco products, is associated with an elevated risk of smell and taste disorders. This finding emphasizes the pressing need for further research and public health interventions that aim to curb tobacco consumption and champion healthy sensory function.

## Data Availability

The data supporting this research are available from the following link: https://www.cdc.gov/nchs/nhis/2021nhis.htm
