# Supplementary material for: Tobacco products and sensory health: An assessment of taste and smell disorders using 2021 NHIS data
Source: Tob Induc Dis. 2024 Feb 8;22:10.18332/tid/181289. doi: 10.18332/tid/181289 (PMC10851189; doi:10.18332/tid/181289)
Supplement: Supplementary file 1 [file TID-22-34-s1.pdf]

Supplementary Table 1: Prevalence of smell, taste, and smell or taste disorders by tobacco use groups among US adults aged 18+ who reported their smoking status and responded to taste and smell questions in the 2021 National Health Interview Survey (NHIS)

Smoking status	Taste disorder (N=29,444)		Smell disorder (N=29,421)		Smell or Taste disorder (N=29,403)	
	%*	95%CI	%*	95%CI	%*	95%CI
**Total US adults**	**9.8**	**(9.3, 10.2)**	**13.4**	**(12.9, 14.0)**	**16.5**	**(15.9, 17.1)**
**Non-tobacco users**	9.5	(9.0, 10.0)	12.9	(12.4, 13.5)	16.0	(15.4, 16.5)
**Cigarettes only**	10.5	(9.2, 11.9)	14.7	(13.1, 16.3)	18.1	(16.3, 19.9)
**E-cigarettes only**	12.3	(9.3, 15.3)	18.5	(14.9, 22.1)	21.1	(17.3, 24.9)
**Cigar, pipe, or smokeless tobacco only**	8.2	(6.5, 10.0)	13.1	(10.9, 15.3)	16.2	(13.8, 18.7)
**Multi-users**	14.6	(11.8, 17.4)	19.1	(16.0, 22.2)	22.5	(19.2, 25.7)

*Weighted percentages
95%CI: 95% confidence interval

Supplementary Table 2: Logistic regression models of associations between smoking status and smell or taste disorders, taste disorders, and smell disorders among NHIS 2021 sample adults.

Smoking	Taste disorder		Smell disorder		Smell or Taste disorder	
	Crude OR (95%CI) (N=29,444)	Adjusted OR (95%CI) (N=29,442)	Crude OR (95%CI) (N=29,421)	Adjusted OR (95%CI) (N=29,419)	Crude OR (95%CI) (N=29,403)	Adjusted OR (95%CI) (N=29,401)
Non-tobacco user	Ref	Ref	Ref	Ref	Ref	Ref
Cigarettes only	1.12 (0.96, 1.30)	1.01 (0.86, 1.18)	1.16* (1.01, 1.33)	1.07 (0.93, 1.23)	1.2* (1.02, 1.32)	1.06 (0.93, 1.21)
E-cigarettes only	1.34* (1.01, 1.76)	1.35* (1.01, 1.78)	1.53* (1.20, 1.94)	1.51* (1.19, 1.93)	1.41* (1.12, 1.76)	1.42* (1.13, 1.80)
Cigar, pipe, or smokeless tobacco only	0.85 (0.68, 1.08)	0.93 (0.73, 1.18)	1.01 (0.83, 1.23)	1.09 (0.89, 1.43)	1.02 (0.84, 1.23)	1.11 (0.92, 1.34)
Multi-user	1.63* (1.29, 2.05)	1.54* (1.22, 1.95)	1.59* (1.29, 1.95)	1.52* (1.22, 1.87)	1.53* (1.26, 1.85)	1.47* (1.21, 1.78)

Adjusted for age, gender, race, and income.
*Indicates statistical significance (p-value <0.05)

Supplementary Table 3: Prevalence and odds ratio of smell and taste disorders by tobacco use groups among US adults aged 18+ who reported their smoking status and responded to taste and smell questions in the 2021 National Health Interview Survey (NHIS)

Tobacco product	Smell and Taste disorder (N=28,503)		Smell and Taste disorder (N=28,503)	
	%[a]	95%CI	Crude OR (95%CI)	Adjusted OR[b] (95%CI)
**Total US adults**	6.7	**(6.3, 7.1)**	-	-
**Non-tobacco users**	6.1	(5.6, 6.6)	Ref	Ref
**Cigarettes only**	6.7	(5.8, 7.6)	1.14 (0.96, 1.35)	1.14 (0.96, 1.35)
**E-cigarettes only**	8.6	(5.2, 11.9)	1.48 (0.96, 2.29)	1.52 (0.96, 2.42)
**Cigar, pipe, or smokeless tobacco only**	6.3	(5.2, 7.4)	1.04 (0.84, 1.28)	1.17 (0.94, 1.44)
**Poly-tobacco product users**	8.0	(7.3, 8.8)	1.40* (1.22, 1.59)	1.48* (1.29, 1.71)

[a] Weighted percentages
[b] Adjusted for age, gender, race, and income.
*Indicates statistical significance (p-value <0.05)

Supplementary Table 4: Logistic regression models of interaction between smoking status and sex on smell or taste disorders, taste disorder, and smell disorder among US adults aged 18+ who reported their smoking status and responded to taste and smell questions in the 2021 National Health Interview Survey (NHIS).

Tobacco product	Taste disorder		Smell disorder		Smell or Taste disorder	
	Crude OR (95%CI) (N=28,483)	Adjusted OR (95%CI) (N=28,483)	Crude OR (95%CI) (N=28,460)	Adjusted OR (95%CI) (N=28,460)	Crude OR (95%CI) (N=28,442)	Adjusted OR (95%CI) (N=28,442)
**Male**						
**Non-tobacco user**	Ref	Ref	Ref	Ref	Ref	Ref
**Cigarettes only**	1.32* (1.03, 1.70)	1.23 (0.95, 1.59)	1.20 (0.95, 1.52)	1.13 (0.89, 1.43)	1.30* (1.04, 1.62)	1.19 (0.96, 1.49)
**E-cigarettes only**	1.68 (0.97, 2.91)	1.75* (1.01, 3.03)	2.15* (1.37, 3.39)	2.17* (1.37, 3.44)	1.83* (1.19, 2.84)	1.88* (1.21, 2.92)
**Cigar, pipe, or smokeless tobacco only**	1.27* (1.00, 1.62)	1.30* (1.03, 1.65)	1.31* (1.06, 1.62)	1.34* (1.08, 1.66)	1.24* (1.02, 1.50)	1.28* (1.05, 1.55)
**Poly-tobacco product users**	1.36* (1.13, 1.63)	1.33* (1.11, 1.60)	1.55* (1.32, 1.84)	1.53* (1.29, 1.81)	1.47* (1.27, 1.70)	1.45* (1.25, 1.67)
**Female**						
**Non-tobacco user**	Ref	Ref	Ref	Ref	Ref	Ref
**Cigarettes only**	1.11 (0.94, 1.32)	1.10 (0.93, 1.31)	1.22* (1.05, 1.41)	1.19* (1.03, 1.38)	1.21* (1.06, 1.38)	1.17* (1.02, 1.34)
**E-cigarettes only**	1.07 (0.69, 1.66)	1.09 (0.69, 1.72)	1.10 (0.75, 1.61)	1.07 (0.72, 1.59)	1.08 (0.76, 1.53)	1.09 (0.75, 1.57)
**Cigar, pipe, or smokeless tobacco only**	0.85 (0.65, 1.11)	0.84 (0.65, 1.10)	0.95 (0.76, 1.20)	0.96 (0.76, 1.20)	0.95 (0.77, 1.18)	0.98 (0.79, 1.21)
**Poly-tobacco product users**	1.58* (1.36, 1.84)	1.49* (1.27, 1.74)	1.53* (1.34, 1.75)	1.43* (1.25, 1.65)	1.56* (1.38, 1.77)	1.48* (1.30, 1.68)

Adjusted for age, gender, race, and income.
*Indicates statistical significance (p-value <0.05)

Supplementary Figure 1: Directed Acyclic Graph (DAG) illustrates the relationship between smoking and smell and taste disorder among the different demographic factors in the model.

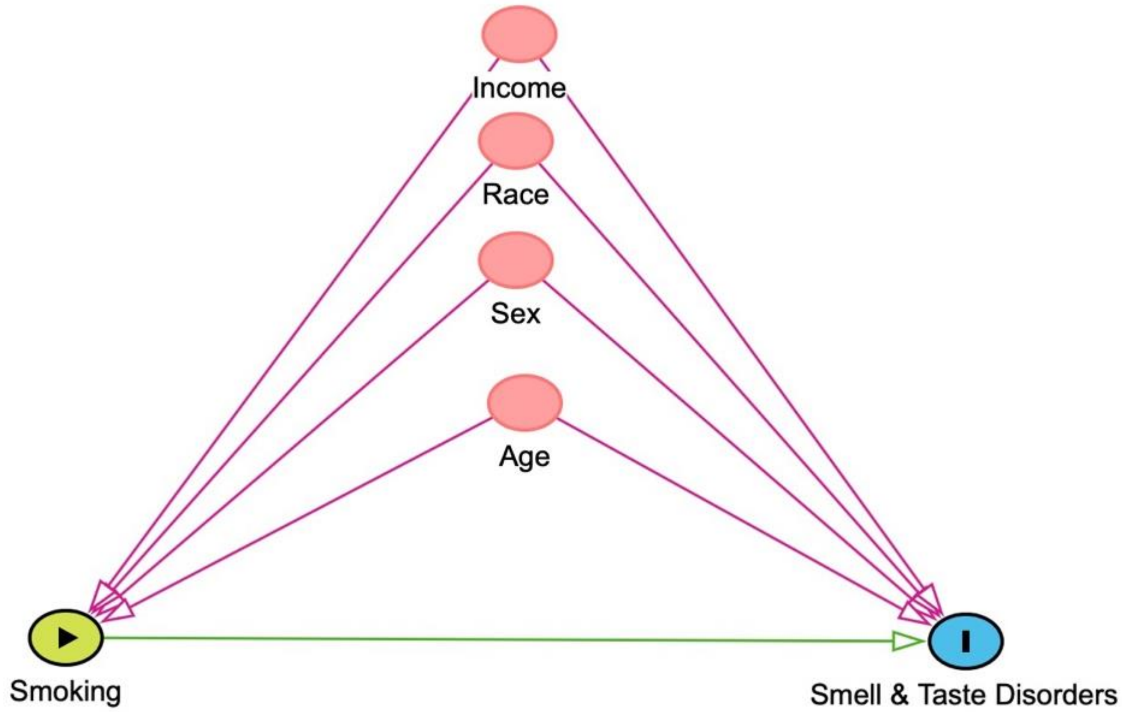

©2024 Bin Hamdan R.H. et al.
